# Beyond canonical homeostasis: Dynamic, plastic, and intermittently unregulated physiology as the norm

**DOI:** 10.14814/phy2.71150

**Published:** 2026-10-19

**Authors:** Warren Burggren, Tobis Wang

**Affiliations:** ^1^ Department of Biological Sciences University of North Texas Denton Texas USA; ^2^ Zoophysiology, Department of Bioscience Aarhus University Aarhus Denmark

**Keywords:** fluctuation, homeostasis, periodicity, regulation, stochasticity

## Abstract

Homeostasis is often taught as steady‐state regulation keeping internal variables within narrow limits—reflecting a mammalian bias. Yet, canonical homeostasis is far from universal. In many animals, including humans, normal physiological states allow some variables to fluctuate cyclically or stochastically while others remain tightly controlled. Indeed, across non‐mammalian species, broad physiological variability is frequently normal rather than exceptional. Here we present case studies showing that plastic, variable regulation—sometimes approaching randomness—is both common and adaptive. Examples include intermittent breathing during diving, episodic feeding and digestion, pulsatile hormone release, variable sleep cycles, irregular gastric emptying, fluctuating renal blood flow, and stochastic immune responses. These complex regulatory patterns represent evolved strategies—not failures of homeostasis—allowing organisms to function efficiently in dynamic environments. Such flexible regulation, including unregulated intervals, may minimize energetic costs compared with continuous tight control, enhancing evolutionary fitness. We propose that variable degrees of physiological regulation have evolved as energy‐efficient solutions shaped by natural selection. Expanding the concept of homeostasis to include intermittent and stochastic forms will improve our understanding of physiological adaptation, with significant implications for ecology and evolutionary biology. We further advocate new experimental approaches to distinguish adaptive variability from true regulatory failure.

## 1 THE CONSTELLATION OF HOMEOSTASIS‐RELATED CONCEPTS

### 1.1 Homeostasis and its ‘offspring’

The classical view of homeostasis—what we call “canonical” homeostasis (pun unintended but unavoidable)—posits that organisms use coordinated regulatory mechanisms to keep key internal variables within a narrow, relatively constant range, despite what can be large changes in the external environment. Almost every paper that evokes the concept of homeostasis starts by paying homage to Claude Bernard and Walter B. Cannon—as they should. Typically, what is then provided is a brief history of the concept of homeostasis—which we will avoid. Rather, we simply direct the reader unfamiliar with the origins and rich history of homeostasis to numerous reviews in this non‐exhaustive list (Bechtel & Bich, 2024a; Billman, 2020; Cooper, 2008; Geary, 2023; Kuang, 2023; Langley, 1973; Modell et al., 2015; Moore‐Ede, 1986; Ruoff, 2026; Woods & Ramsay, 2007; Woods & Wilson, 2013; Woods & Wilson, 2014). Notably, the classical concept of homeostasis has begat numerous “conceptual children”—allostasis, adaptive homeostasis, hormesis, heterostasis, etc. (Table 1). These emergent concepts are attempts to explain the back‐and‐forth tug many animals experience between varying internal and environmental stressors on the one hand, and the perceived need to relatively precisely control the *milieu intérieur* on the other. This dynamic is fully realized in the term “adaptive homeostasis”, whereby the range around the setpoint is adjusted in response to signaling molecules or events (Davies, 2016; Davies, 2018; Zastrow et al., 2025). Similarly, “allostasis”, “heterostasis”, “hormesis”, and related terms expand upon the concept of physiological regulation in a dynamic environment with multiple and/or variable stressors (Ruoff, 2026; Schulte, 2014).

**TABLE 1:** Homeostasis and its derivative terms. Terms proposed in the literature are presented in order of first appearance in the literature on physiological regulation. Notably, these definitions have been reiterated multiple times since their first introduction into the literature. Here we offer our synthesis of these various explanations.

Term	Proposing author(s) and date	Definition (quoted from original proposing article)	Description
Homeostasis	Cannon (1932)	“(Homeostasis is) …the coordinated physiological processes which maintain most of the steady states in the organisms (…) involving, as they may, the brain and nerves, the heart, lungs, kidneys and spleen, all working cooperatively”	Cannon (1932) builds on Claude Bernard's concept of milieu intérieur, and describes mechanisms for maintaining internal constancy, especially in mammals and in humans in particular.
Hormesis	Southam and Ehrlich (1943)	“The term hormesis (adj. hormetic) is proposed to designate such a stimulatory effect of subinhibitory concentrations of any toxic substance on any organism.”	Hormesis as originally defined by Southam and Ehrlich (1943) refers to a biphasic dose–response to a stressor or toxin. Low doses of the stressor generate a beneficial adaptive effect, while high doses result in inhibitory or toxic effects.
Homeorhesis	Waddington (1957)	We need, therefore, three separate notions in connection with biological tendencies towards equilibria: physiological homeostasis, when it is some physiological state which is being held constant; development homeorhesis or canalisation when it is the course of development which is relatively invariant; and genetic homeostasis when the constant feature of the system is its set of gene frequencies.	Homeorhesis refers to the tendency of a developing system to return to a characteristic trajectory of change, rather than to a fixed state suggested by homeostasis. Waddington generally equates his homeorhesis with his concept of canalization.
Heterostasis	Selye (1973)	“(Heterostasis is)…establishment of a new steady state by exogenous (pharmacologic) stimulation of adaptive mechanisms through the development and maintenance of dormant tissue reactions.”	Heterostasis describes control systems/situations where regulatory mechanisms with lower feedback gain allow for more variable responses to disturbances. Heterostasis is often contrasted with homeostasis, when in fact that really just differ by the range between upper and lower setpoint values.
Enantiostasis	Mangum and Towle (1977)	“Another form of physiological regulation also permits freedom from the simple consequences of environmental change. We propose to designate this kine of adaptation as enantiostasis, a word composed of three Greek elements meaning a counterbalanced state of opposing conditions.”	Enantiostasis is the capacity of an organism to maintain effective metabolic and physiological function while external conditions and associated internal variables change, prioritizing sustained functionality rather than fixed internal states under variable environments.
Predictive and reactive homeostasis	Moore‐Ede (1986)	“A mature understanding of homeostasis should encompass both “reactive” responses to changes in physiological variables which have already occurred and the “predictive” responses initiated in anticipation of predictably timed challenges.”	While canonical homeostasis describes how physiological systems react to environmental stressors to maintain the milieu intérieur, predictive homeostasis describes physiological adjustments in advance of anticipated, predictably timed environmental stressors.
Allostasis	Sterling and Eyer (1988)	“Allostasis emphasizes that the internal milieu varies to meet perceived and anticipated demand. This variation is achieved by multiple, mutually reinforcing neural and neuroendocrine mechanisms that override the homeostatic mechanisms […] emphasizing the subordination of local feedbacks to control by the brain”	Allostasis, like heterostasis (above), expands, rather than as sometimes argued, modifies the concept of homeostasis. Allostasis allows for varying internal milieu that nonetheless is highly regulated by neural and endocrine mechanisms.
Rheostasis	Mrosovsky (1990)	“Rheostasis refers to a condition or state in which, at any one instant, homeostasis defenses are still present but over a span of time there is a change in the regulated level. … rheostasis includes a change in set‐point, both when the term is used descriptively without specifying a mechanism…. And when it is used to indicate a mechanism comprising negative feedback with a reference signal.	Although several authors discussed the concept of physiological regulation during changing external conditions, Mrosovsky was the first to coin and define the term “Rheostasis” involving a change in set point for a regulated variable.
Homeodynamic regulation	Dworkin (1993)	Authors' Note: Although widely attributed to Dworkin (1993), the term homeodynamic regulation is not listed in the index nor identifiable in the text of the book	Homeodynamic regulation was proposed before introduction of the term allostasis (see above). Both refer to variation in physiological regulation and associated set points that maintains the internal milieu in the face of fluctuating environmental conditions.
Adaptive homeostasis	Davies (2016)	“(Adaptive homeostasis is) …The transient expansion or contraction of the homeostatic range in response to exposure to sub‐toxic, nondamaging, signaling molecules or events, or the removal or cessation of such molecules or events.”	In defining Adaptive Homeostasis, Davies (2016) emphasizes how low and high set points are, in fact, not set, but rather expand and contract as chemical environmental stressors wax and wane.
Temporal homeostasis	Perreau‐Lenz et al. (2004)	Authors' Note: While no formal definition is provided in the indicated journal article, by implication temporal homeostasis is a homeostatic state resulting “…from a diverse set of biological clock outputs”.	“Temporal homeostasis” or regulated rhythmic variability, in which physiological regulation to certain setpoints is adjusted according to external environmental conditions

These derivative terms typically emphasize the type of response under various scenarios used to maintaining relative constancy of the *milieu intérieur*—all through frequent if not constant adjustments in the physiological control system. A major differentiating factor between members of the constellation of homeostasis‐related concepts presented in Table 1 involves a dynamic element—that is, how rapidly and profoundly do these proposed regulatory phenomena respond to variable environmental stressors and how rigorously is the *milieu intérieur* defended?

We emphasize that most of the variations on regulatory control in Table 1 provide an effective framework for describing regulatory processes when the end goal is maintaining relatively constant internal variables (e.g., blood pH, body temperature, and tissue oxygenation) in the face of fluctuations in internal or environmental stressors. Here, we argue here that the current constellation of homeostatic terms is conceptually limited in describing physiological regulatory processes and patterns when physiological regulation is normally characterized by generally high plasticity, which can include: (1) extremely wide setpoint ranges, (2) stochastic, intermittent, and unregulated swings of the internal milieu, (3) highly periodic or intermittent physiological processes that may even temporarily cease, and (4) even seeming temporary abandonment of physiological regulation. Indeed, for some processes, in some animals, there can be an intermittent, periodic, or irregular apparent lack of physiological regulation lasting from minutes to hours or even days. Such highly plastic non‐steady state or even intermittent physiological regulation is rarely the focus of discussion of homeostasis broadly writ, and consequently is the focus of this article.

Also important to emphasize is that an adaptive plastic homeostasis is distinct from failure to regulate physiological processes resulting from pathologies. Too often wide variation in presumably well‐regulated physiological processes is viewed through the lens of disease through so‐called overdiagnosis (Brodersen et al., 2018; Rogers & Mintzker, 2016). In fact, wide variation in the absence of pathology can be attributed to variability resulting from genetic or other intrinsic factors (Birch, 2006; Herold et al., 2021; Price et al., 2003). Of course, differentiating between normal versus pathological variations can be problematic, whether assessing humans or starfish. While beyond the scope of this article to provide such guidance, we suggest the first step begins with recognizing how plastic homeostasis can be.

### 1.2 Goals of our review

We emphasize at the outset of our review that we are not proposing yet another term to describe some variant on homeostasis—our Table 1 is large enough! Nor are we claiming novelty by indicating that physiological variation is the norm. Indeed, this has been understood for a very long time and frequently re‐emphasized through both observations in the laboratory and teaching in the classroom (Collins, 1999; Himmelstein et al., 1990; Lewontin, 1983; Prosser, 1955; Schmidt‐Nielsen, 1997; Spicer & Gaston, 1999/2000; Turner, 2010; Weiner, 1977). Instead, we are “simply” saying that at certain times (often brief), under certain circumstances (season, reproductive condition, prandial state etc.), in certain species (invertebrates and vertebrates including humans), there appears to be no homeostatic regulation in effect.

Much of what comprises normal variation versus pathology comes from a mammalian bias in physiological studies, as we now explore.

## 2 MAMMALIAN BIAS INFILTRATES THE CONCEPT OF HOMEOSTASIS

The classic view of physiological regulation involves very well understood and documented regulatory mechanisms in animals with a relatively narrow range of upper and lower setpoints for the regulated variable (Billman, 2020; Geary, 2023). For better or worse, this view is driven by the heavy dominance of mammalian physiology in the literature of physiological regulation. Underscoring this notion, an informal PubMed search we conducted in June 2026 for literature trends reveals that, of the ~440,000 publications containing the word “*homeostasis*”, nearly 80% are partially or exclusively based on mammals. Thus, whether considering homeostatic regulation of blood pressure, body temperature, plasma pH, or numerous other biological variables, there is an advertent bias—both in terms of the number of available publications and the perspective of researchers—towards investigation of those processes in which these variables are typically tightly regulated within narrow limits (Fernández‐Peña et al., 2023; Loftus et al., 2022; Proctor et al., 2013). Not surprisingly, then, medical students, for example, are taught that healthy humans (and all other mammals but for what are presented as weird oddities) typically maintain blood oxygen, carbon dioxide, bicarbonate, and pH within a fairly narrow set of limits (Aoi & Marunaka, 2014; Atherton, 2009; Cummins et al., 2020; Maltepe & Saugstad, 2009; Tretter et al., 2020). Minute‐to‐minute variations are typically modest, while large excursions over time in normally tightly controlled variables generally indicate a pathology. Yet, under this veil of perceived constancy in humans (and by implication other mammals), there are a surprising number of physiological processes and variables that vary widely in space and time—sometimes rhythmically, and sometimes stochastically. Thus, there is a dual bias—a slant towards mammals plus a focus on tightly regulated variables wherever they occur.

We posit here that the mammalian‐centric focus on relative homeostatic constancy of the *milieu intérieur* only obliquely applies to the many animals with intrinsic, large variation in physiological regulation as part of their natural biology. Physiologists studying invertebrates typically view such large variation as the norm rather than an anomaly or pathology (Bechtel & Bich, 2024b; Chown, 2001). These same researchers would argue that homeostasis “belongs” as much to these animals as to the mammals to which it was originally attributed (Bechtel & Bich, 2024a). Even vertebrates with tight homeostatic regulation that keep physiological variables within narrow ranges (e.g., most birds and mammals) have specific physiological processes that are at certain times loosely regulated or even unregulated due to their intermittency. Put differently, physiological regulation may vary over time, in response to different environments and when in different physiological states, as we now explore through a series of case studies.

## 3 HIGHLY PLASTIC REGULATION OF PHYSIOLOGICAL PROCESSES – CASE STUDIES

“Maintaining physiological variables within narrow operating limits by homeostatic mechanisms is a fundamental property of most if not all living cells and organisms (emphasis ours)” (Swann & Rho, 2014). This well‐intentioned (over)statement, derived from a biomedical paper addressing homeostasis in human patients, sets aside the many—perhaps the majority—of non‐human species in which physiological variables are not maintained within narrow operating limits, and indeed may at times be more or less unregulated. Such variation frequently is environmentally‐driven—for example, weather and food availability. Alternatively, there can be “intrinsic” non‐pathological variability of physiological variables that are dialed in to the species' normal physiology—for example, gastric emptying and pulsatile endocrine secretion.

As demonstration of this point, we now develop several example “case studies”, drawn from both medical and comparative physiology, based on whether the source of variation in the *milieu intérieur* was environmentally driven (Section 3.1) or more intrinsic (Section 3.2).

### 3.1 Environmentally‐driven regulatory intermittency

#### 3.1.1 Case study #1: Thermoregulation in variable environments

Constancy of body temperature is a stalwart of classic homeostasis as it applies to mammals and especially humans. Variations of more than a few ^o^C in humans are attributed to a pathological condition, often associated with malaria, septicemia, etc. (Guzelj et al., 2024; Kiyatkin, 2019; Koyama et al., 2024; Kushimoto et al., 2014; Maloney & Duffy, 2024). Yet, healthy humans are somewhat exceptional among mammals in maintaining a relative constancy of body temperature. Many mammals—both wild and domesticated—inhabiting temperate climates may show relatively large swings in body temperature associated with fluctuations in environmental conditions (Mitchell et al., 2018; Piccione et al., 2003; Riek et al., 2017; Russel et al., 2025). This so‐called “adaptive heterothermy” occurs in many taxa with which we typically associate regulation of body temperature within narrow limits (Auteri, 2022; Bastos et al., 2021; Boratyński et al., 2024; Boyles et al., 2011; Levesque et al., 2023; Mitchell et al., 2002; Mitchell et al., 2018; Morales et al., 2021; Mota‐Rojas et al., 2021). Heterothermy can be a daily event, or can be tied to decreases in food availability, prolonged environmental temperature stress, etc. (Bastos et al., 2021; Boratyński et al., 2024; Levesque et al., 2023; Morales et al., 2021).

Flying in the face of the concept of carefully maintained specific body temperatures are the large fluctuations in body temperature that can be associated with torpor, winter sleep, hibernation, and estivation in vertebrates (Geiser, 2008; Geiser, 2020; Giroud et al., 2020; Kontopoulos et al., 2025; Shankar et al., 2023) and invertebrates (Bale & Hayward, 2010; Marshall & McQuaid, 2020; Rodriguez et al., 2023). Such variations in body temperature, which often have apparently stochastic components, arise from complex interactions of endogenous clocks, metabolic regulation, and environmental conditions (Brodin et al., 2017; Mota‐Rojas et al., 2021).

What characterizes many animals that are highly tolerant of major body temperature fluctuations, as well as variations in metabolic rate and timing of arousal episodes, is that when in moderate thermal environments they nonetheless show regulatory capacities that suggest homeostatic regulation of physiological variables within precise, narrow ranges. Indeed, some researchers assert that physiological regulation during hibernation, for example, is an extension of homeostasis found during activity with warmer body temperatures (Arfat et al., 2020; Boyer & Barnes, 1999; Drew et al., 2007; Junkins et al., 2022). Noteworthy, however, is that this view often results from parsing out and focusing on particular regulated variables—for example, calcium levels, blood volume, hemostasis, etc.—often to the exclusion of other poorly or apparently non‐regulated variables. This highlights an important point—homeostasis is not all‐pervasive, which would be reflected in the equivalently effective regulation of all possible physiological variables. Rather, animals that at some point typically show wide variations in physiological variables may show “selective homeostasis”, maintaining some variables but not others.

#### 3.1.2 Case study #2: Intermittent gas exchange

Independent of environmental influences, breathing and thus gas exchange with the environment can be highly intermittent in many animal taxa (including marine mammals). Intermittent ventilation, characterized by periods of apnea measured in many minutes if not hours, naturally occurs in diving mammals and birds—for example, Burggren et al. (2024), Blawas et al. (2021), and Panneton and Gan (2020). Both diving and terrestrial reptiles experience intermittent breathing, which is also the norm in aquatic amphibians, diving birds, and most airbreathing fishes (Fonseca et al., 2012; Lefevre et al., 2016; Mendez‐Sanchez & Burggren, 2014; Taylor et al., 1999). Accordingly, blood and tissue pH and oxygenation and can vary over an extreme range compared to constantly breathing animals (Burggren et al., 2024; Fahlman et al., 2024; Williams & Davis, 2021). As just a few examples, diving freshwater turtles may show excursions of more than 100 mmHg in arterial blood PO_2_ during unusually long voluntary dives of nearly 3 h (Burggren & Shelton, 1979), while in captive dolphins during voluntary prolonged apnea blood PCO_2_ increases from 54 to 84 mmHg and concurrently blood pH drops from 7.54 to 7.35 (Terasawa et al., 2010). Equally drastic swings in blood and hemolymph respiratory gases and pH can occur during intermittent air breathing in air breathing fishes and invertebrates that show intermittent ventilation (Graham, 1997; Matthews & White, 2011; Randall et al., 1981).

Importantly, such observations do not mean that intermittently breathing animals lack homeostatic mechanisms for regulating respiration‐related physiology. Rather, such animals (1) have an enhanced tolerance to very wide variations in their *milieu intérieur* compared to constantly breathing animals, and/or (2) have highly plastic regulatory mechanisms that can “ignore” internal setpoints for blood chemistry, relying upon other mechanisms for stimulating resumption of breathing.

#### 3.1.3 Case study #3: Intermittent feeding and digestion

Animals that feed intermittently, exemplified by large snakes like pythons and boas, often alternate between prolonged fasting and infrequent, massive meals, often 20%–100% of the snake's total body mass (Last et al., 2025; Rindom et al., 2025). Notably, the absence of the gene for ghrelin—the “hunger hormone”—prevents hunger signals that might otherwise drive predation or increase metabolic rate (Pinto et al., 2026). Lack of ghrelin‐driven hunger decouples foraging from energy deficit signals, allowing snakes to endure months of nutritional disequilibrium without behavioral distress. Such animals exhibit remarkably plastic homeostatic regulation related to digestion, internal nutrient transport, and metabolism (Holmberg et al., 2002; Last et al., 2025; Lillywhite, 2017). During fasting periods, which can last weeks or even months, fasting snakes drastically downregulate their metabolism to conserve energy, relying on fat reserves without the rapid fat breakdown seen in mammals during fasting (McCue, 2007). Concurrently, fasting snakes may experience a reduction in mass of 50%–80% through tissue shrinkage (and associated functional reduction) in organs like the heart, intestines, liver, and kidneys (Ott & Secor, 2007; Secor et al., 2012; Secor & Diamond, 1995; Tan et al., 2023). This atrophy minimizes energy expenditure that, in part, would be expended on tissue maintenance and function.

Upon ingesting prey, these intermittent feeders begin to show a specific dynamic action (SDA) that is among the most profound of all animals (Figure 1). Metabolism can increase as much as 10–40‐fold, enabled by a dramatic hypertrophy of the visceral organs hypertrophy, reversing starvation's longer‐term effects on organ mass and function (Bury, 2021; Last et al., 2024; McCue, 2006; McCue et al., 2005). These changes are extraordinarily rapid, with the small intestine potentially doubling in mass within the first 48 h following feeding. Not surprisingly, there is a tremendous upregulation of nutrient transporters, digestive enzymes, and blood flow to facilitate breakdown of ingested tissues, which may include bones, fur, and other relatively indigestible tissues (Andrew et al., 2015; Secor et al., 1994). Cardiovascular adjustments, including heart enlargement and greatly elevated cardiac output, support the aerobic demands of digesting the newly eaten meal (Jensen & Wang, 2024), while hydration needs increase, prompting postprandial drinking despite metabolic water from prey (Lillywhite, 2017).

**FIGURE 1:**
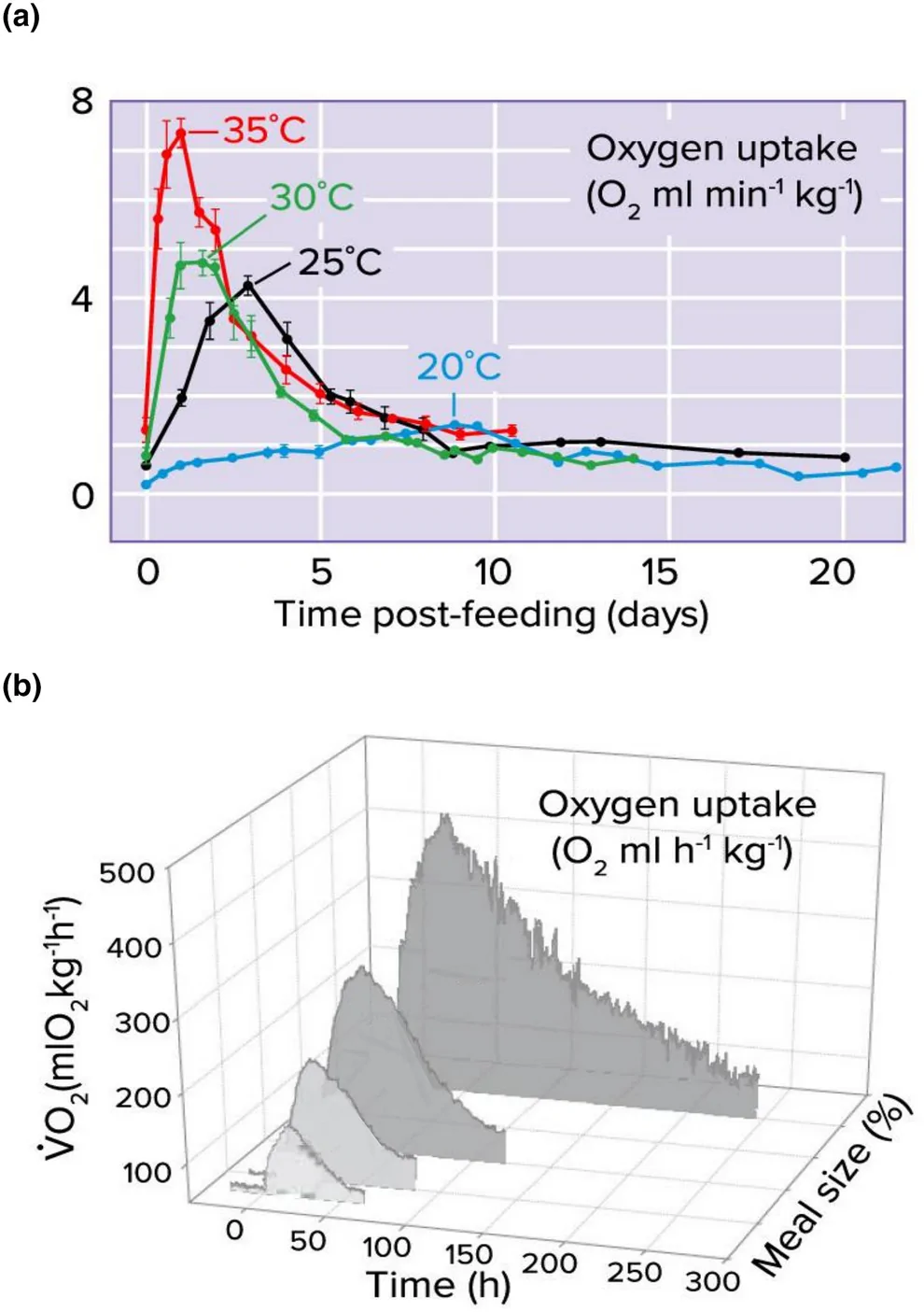
Specific dynamic action in the Urutu pit viper (Bothrops alternatus) as a function of both temperature and meal size. (a) Temperature‐dependency of the SDA response. (b) The effect of meal size on the SDA response. From Jensen and Wang (2024).

The highly cyclic and reversible anatomical, physiological and metabolic plasticity of large predatory snakes and other intermittent feeders does not reflect the canonical steady‐state model so much as it represents rheostasis (Table 1), with these animals prioritizing survival through extreme, cyclic set‐point shifts. While evidence to data suggests that the digestive process remains regulated at least in snakes, clearly there are enormous adjustments in physiological set points during such prolonged fasting. True steady‐state homeostasis in the classic sense likely emerges only briefly post‐digestion or deep into fasting; most of the cycle involves controlled deviation from mammalian norms, highlighting evolutionary trade‐offs for infrequent feeding.

#### 3.1.4 Case study #4: Diel, tidal/lunar, and seasonal metabolic rhythms

The large majority of organisms exhibit physiological rhythms to some degree associated with diurnal, tidal, or lunar rhythms (Coskun et al., 2023). Most common are circadian rhythmicity of regulated body temperature—see (Morin et al., 2025; Refinetti, 2020). However, to clearly demonstrate physiological rhythms that are intrinsic, rather than “simply” driven by the internal or external environment—especially body temperature changes—is a non‐trivial task (Beale et al., 2016).

Diurnal changes have been well documented in metabolic rate (Begemann et al., 2025; Ruddick‐Collins et al., 2022) and the associated metabolome (Fatima & Rana, 2020; Hancox et al., 2021; Preußner et al., 2017). However, reports for humans of metabolic rate changes that are independent of body temperature are relatively rare, and most typically are associated with activity or pathology. Even then, cause and effect between small body temperature changes and adjustments in metabolic rate in humans are difficult to determine (Refinetti, 2020). Yet, many mammals (including domesticated animals and popular experimental animals such as rodents) as well as birds routinely show clear temperature‐independent metabolic rhythms (Refinetti, 2020). In many squamate reptiles, metabolic rate increases at sunrise in the absence of any external cues such as light or changes in temperature (Dubiner et al., 2023), as measured in dozens of lizard species in Israel (Dubiner et al., 2023). The epaulet shark (*Hemiscyllium ocellatum*) shows nearly two‐fold metabolic rate diel rhythms independent of body temperature (Wheeler et al., 2022). Invertebrates such as insects also show diurnal changes in metabolic rate (Stahlschmidt, 2022), with intrinsic pulsation and rhythms in expression of genes associated with metabolism (Li et al., 2025). Diurnal rhythmicity of microbes is also well established (Heddes et al., 2022; Wang et al., 2022), suggesting that such rhythms are a basic construct across the tree of life.

The innate basis for tidal rhythms in metabolism and other processes has long been established (Palmer, 1973). However, our ability to truly isolate experimental animals from all possible tidal/lunar zeitgebers remains questionable (de Mello Gallep & Robert, 2022). Nonetheless, investigation of both tidal and lunar‐controlled metabolic rhythms (Horn et al., 2021), as well as their molecular and genetic basis, continues to be actively investigated (Andreatta & Tessmar‐Raible, 2020; Oliphant et al., 2025).

Seasonal rhythms in metabolic rate independent of body temperature are said to be lacking in humans (Anthanont et al., 2017). In other mammals the many‐fold reduction in metabolic rate during winter sleep, torpor, or hibernation is well documented—see (Giroud et al., 2020; Scopes et al., 2024). While these decreases in metabolism are often intertwined with decreases in body temperature, there is evidence of the independence of the two (Swoap, 2008). Outside of the specialized metabolic states of torpor, winter sleep, and hibernation, metabolic rate seasonal rhythms independent of body temperature are apparent in squamate reptiles, where there can be a reduced metabolic rate in “winter” compared to ‘summer’ lizards measured under identical conditions (Figure 2) (Milsom et al., 2008). Similar findings of persistent metabolic rhythms exist for anuran amphibians (Chang & Lucy Hou, 2005) and fishes (Speers‐Roesch et al., 2018; Warren‐Myers et al., 2021).

**FIGURE 2:**
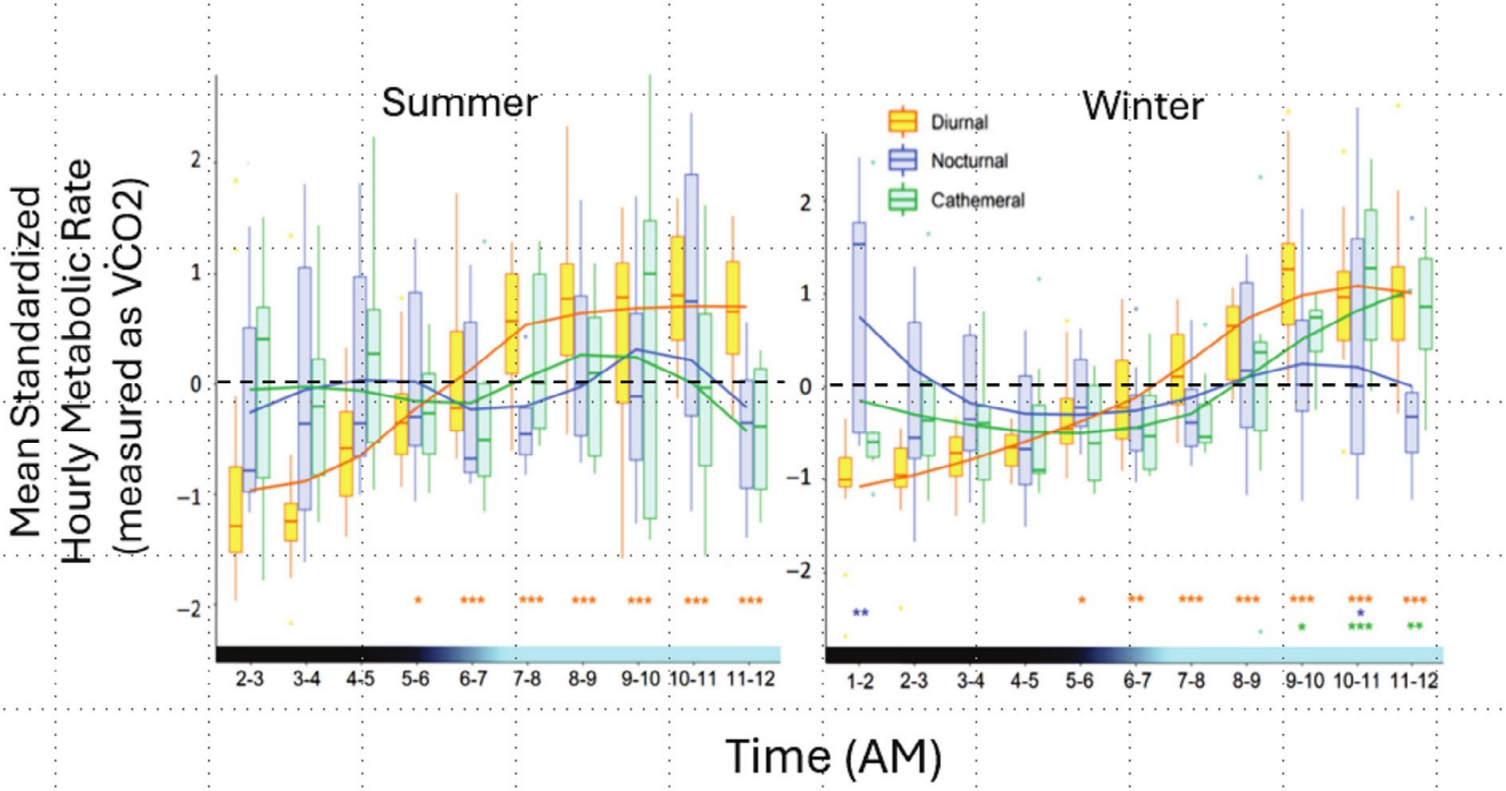
Intrinsic variation in hourly resting metabolic rate in Israeli lizard species. Solid lines represent mean CO_2_ production for diurnal, nocturnal, and cathemeral species. Resting metabolism varied with hour of the day during both summer and winter. Squamate metabolic rates decrease in winter beyond the effect of temperature *p < 0.05, **p < 0.01, ***p < 0.001. From Dubiner et al. (2023).

To summarize, these four Case Studies presenting examples of rhythms in physiological variables suggest a “temporal homeostasis” or regulated rhythmicity, in which physiological regulation to certain setpoints is adjusted according to external environmental conditions (Perreau‐Lenz et al., 2004; Rosenberg et al., 2023). Rather than indicating a lack of homeostatic regulation, molecular, genetic, and metabolic feedback loops maintain an appropriate, condition‐specific level of physiological regulation, albeit highly plastic.

Having discussed variations in physiological regulation that depart from the canonical homeostatic regulation due to environmental stressors, we now turn to variations in physiological variables apparently deriving from intrinsic regulatory elements.

### 3.2 Internally‐driven (intrinsic) regulatory intermittency/stochasticity

#### 3.2.1 Case study #5: Intermittent, pulsatile endocrine secretion

The highly pulsatile release (and thus action) of numerous hormones in mammals has long been appreciated (Carnes et al., 1988; Dierschke et al., 1970; Evans et al., 2009; Veldhuis et al., 1994; Veldhuis et al., 2008). A classic example in mammals is the secretion of gonadotropin‐releasing hormone (GnRH) and then, in turn, the secretion of luteinizing hormone (LH), both secreted in a highly pulsatile manner (Ackland et al., 1994; Dierschke et al., 1970; Han et al., 2020; Herbison, 2018). Regular, rhythmic pulsing of GnRH to trigger LH release is a characteristic of female mammals. The frequency of the various secretory components of the triggered LH pulse, as well as the amplitude of the resulting plasma LH concentration, varies nearly two‐fold among humans, monkeys, and sheep (see Table 1 in Herbison (2018)). Other hormones in mammals that are released in a highly periodic fashion of 10–20 pulses per day include growth hormone (Dickson, 2022; Norman et al., 2013), insulin (Laurenti et al., 2021; Pørksen, 2002), glucagon (Veldhuis et al., 2008), thyroid‐stimulating hormone (van der Spoel et al., 2021), adrenocorticotropic hormone (Faghih et al., 2015; Gudmundsson & Carnes, 1997), glucocorticoids (Roelfsema et al., 2016; Scheff et al., 2012), and parathyroid hormone (Chiavistelli et al., 2015). The pulsatile release of many of the hormones indicated above, while a normal part of daily endocrine regulation. That pulses are irregularly spaced and of variable amplitude is inconsistent with a simple deterministic pacemaker, likely reflecting noisy or stochastic oscillators (Filicori, 2023).

Beyond “simple” pulsatility of hormone excretion, there are also well documented instances in both health and disease where the periodicity and amplitude of hormone release is actually stochastic (Keenan et al., 2003; Veldhuis et al., 2008; Zhang et al., 2025). Such stochastic hormone release can occur as intermittent bursts as part of more complex rhythmic patterns, and result from biological factors (e.g. stress, nutrition, age, and metabolic state) and hormone receptor desensitization or facilitation between hormone pulses (Navarro, 2012). Reflecting the unpredictable nature of aspects of hormone release, the stochastic elements of hormone pulsatility have required complex modeling to fully understand (Brown et al., 2001; Keenan & Veldhuis, 1997; Veldhuis et al., 2008; Zhang et al., 2025). Figure 3 shows irregular timing of LH pulses in female mice. While the timing of LH release might be termed “pulsatile” and even “irregular”, the concomitantly great variation in plasma concentration highs and lows strongly suggest a stochastic component to such release.

**FIGURE 3:**
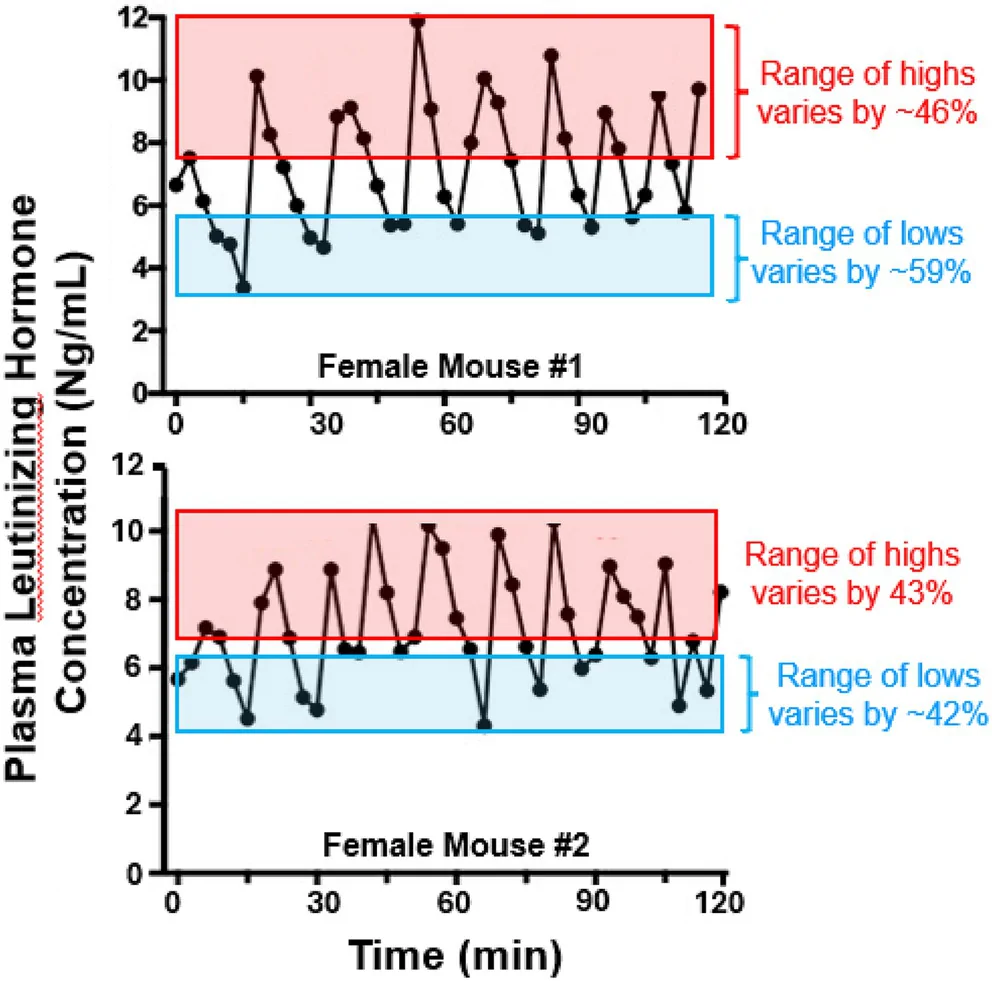
Endogenous pulsatility in plasma luteinizing hormone concentration in two ovariectomized female C57B6 mice. Indicated to the right is the variation in the high and low concentrations over a 2‐h period. Similar large variation occurs in the frequency of luteinizing hormone pulses. Modified from Campos and Herbison (2014).

Notably, some endocrine time‐series studies describe such variation in time and magnitude of hormone secretion as “irregular” or “noisy” without using the word “stochastic”. Whatever we choose to call it, such patterns of hormone release do not readily fit into the framework of classical homeostasis. In that framework, hormones would be released according to negative feedback loops that maintain hormone levels within a relatively narrow range, or in the case of sex hormones, according to a narrow temporal pattern of hormone concentration change. Irregular or stochastic hormone pulses fit rather poorly into the homeodynamic regulation or allostasis framework. Notably, in this scenario, physiological regulation, per se, involves *accommodating* the irregular, stochastic hormone pulses, rather than modulating the pulses themselves.

#### 3.2.2 Case study #6: Sleep and sleep cycles

“Sleep homeostasis” refers to the drive to sleep balanced against fatigue accumulation (Foster, 2020; Nollet et al., 2023; Norman et al., 2024; Thomas et al., 2020). Certainly, the overall homeostatic regulation of sleep has been extensively documented (Dik et al., 2014; Dredla & Braley, 2025; Jiao et al., 2025; Norman et al., 2024). Sleep is highly phasic in humans and other mammals (Dimanico et al., 2021; Nunn et al., 2016; Reinhardt et al., 2019; Simor et al., 2020). While sleep contains rhythmic elements (sleep cycles), there are also intermittent, non‐rhythmic events (e.g., K‐complexes, sleep spindles, and microarousals). These “sleep phasic events” do not follow a strict periodicity and indeed show considerable unpredictable appearances during sleep (Figure 4) (Barbato & Wehr, 2025; Mogavero et al., 2025; Rosenblum et al., 2025; Thomas, 2025).

**FIGURE 4:**
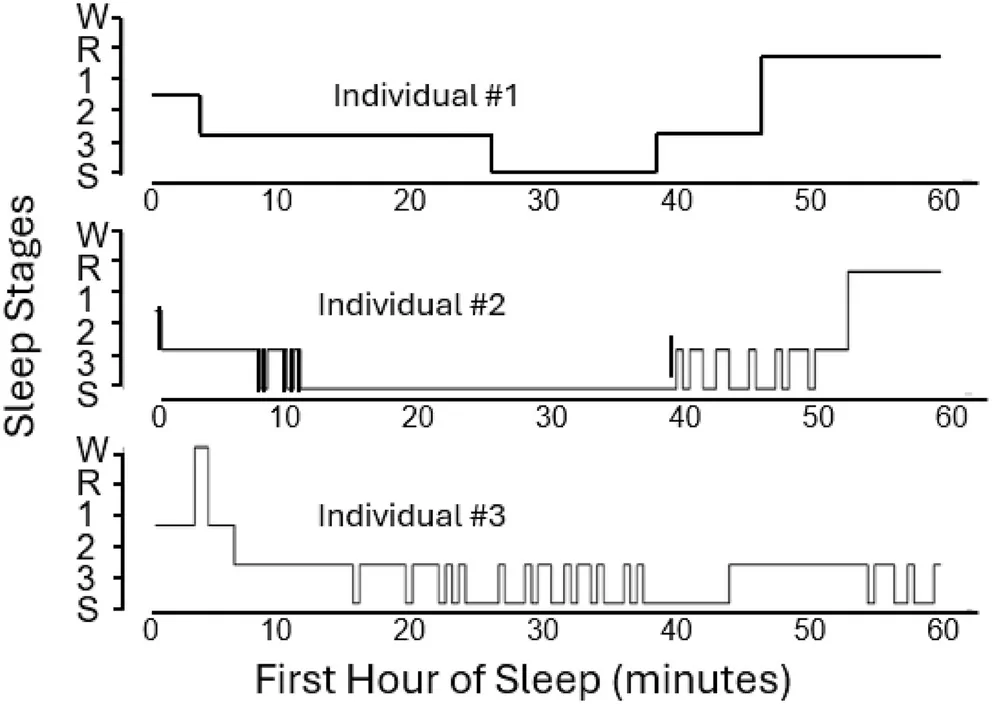
Variation within and between individuals in the various stages of sleep and their duration in three humans. Modified from Swihart et al. (2015).

Sleep is, of course, a broad phenomenon within the animal kingdom, though not all lower vertebrates exhibit the typical sleep cycles of mammals—see Libourel and Herrel (2016). While sleep homeostasis has been described for several fish species; for example, sharks appear to lack classic sleep homeostasis (Kelly et al., 2019; Norman et al., 2024).

#### 3.2.3 Case study #7: Renal blood flow intermittency

Glomerular filtration is frequently presented as an example of a relatively well‐regulated system operating within a normal physiological range of variables despite afferent and efferent arteriole vasodilation or vasoconstriction altering glomerular blood pressure and flow. In reality, “Renal vascular control mechanisms affecting glomerular function do not simply react to physiological challenges, but exhibit spontaneous and dynamic changes” (Holstein‐Rathlou & Marsh, 1994). Indeed, there can be pronounced periodic and irregular oscillations in tubuloglomerular blood flow, blood pressure and feedback control (Holstein‐Rathlou & Leyssac, 1986; Holstein‐Rathlou & Marsh, 1994) (Figure 5). As Peti‐Peterdi and colleagues state, their imaging techniques “…helped to visualize what most physiology textbooks avoid discussing (emphasis ours), namely the dynamic temporal variations in individual nephron and cell functions.” (Peti‐Peterdi et al., 2015). In humans, these blood flow fluctuations can be correlated with factors such as exercise, circadian rhythms, diet, or body temperature (Alhummiany et al., 2024). Clearly, general levels of renal blood flow and function are maintained through homeostatic mechanisms in the healthy kidney. However, at the level of various regions of the kidney itself—and perhaps even at the level of individual nephrons—there is a great variability in blood flow in both time and space that lies outside of canonical homeostatic regulation (Holstein‐Rathlou et al., 2011; Holstein‐Rathlou & Marsh, 1994; Postnov et al., 2022).

**FIGURE 5:**
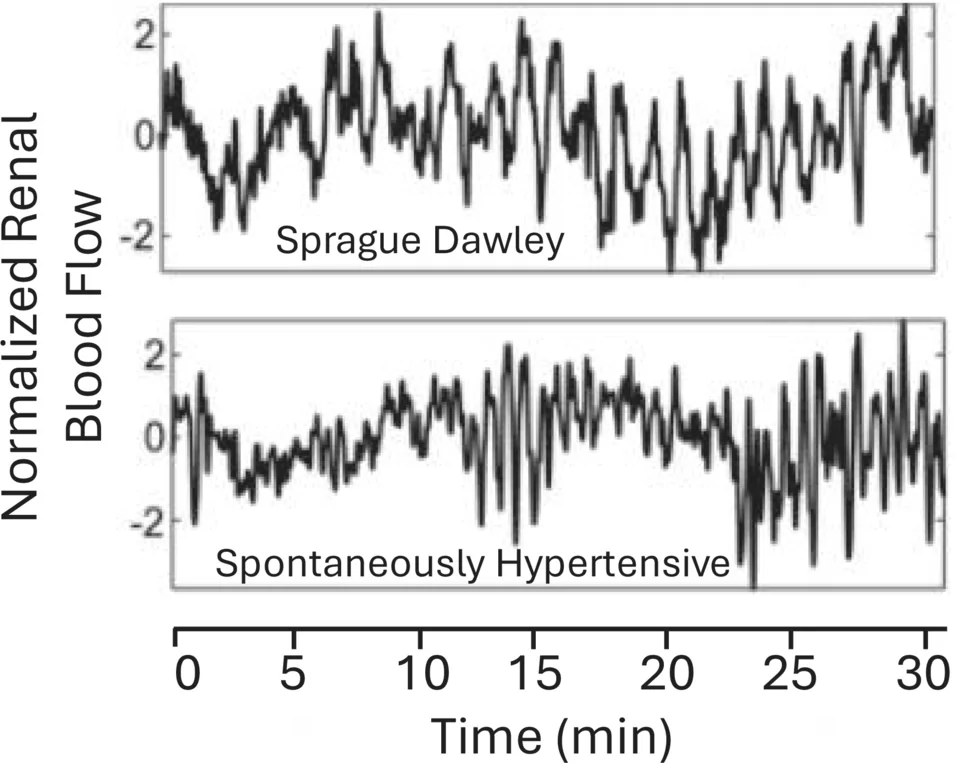
Irregular blood in vivo renal blood flow in Sprague Dawley and spontaneously hypertensive rats. Both minute‐to‐minute and even longer‐term oscillations occur, likely related to stochastic changes in blood flow at the nephron level. Modified from Lee et al. (2023).

#### 3.2.4 Case study #8: Gastric emptying

Gastric emptying in mammals involves clearly identified phases—for example, filling, pumping, lag, and linear emptying phases. The control of gastric emptying is quite complex, involving the actions of at least a dozen hormones and even more classes of neurons (Goyal et al., 2019). While the phases of gastric emptying are distinct, the actual pattern of gastric emptying is often characterized as irregular (Camilleri et al., 2023; Cassilly et al., 2008; Chang et al., 1997; Stotzer & Abrahamsson, 2000). Some of the variability in gastric emptying in mammals stems from differences in stomach contents, but even controlling for standardized meals there is intrinsic variation in both phase duration and intensity of contraction within individuals over time (Camilleri et al., 2023).

Gastric emptying in non‐mammals is much less understood, although many of the same control mechanisms as in mammals are evolutionarily conserved, having been identified in other vertebrates such as reptiles (especially intermittently feeding reptiles) (Holmberg et al., 2002; Secor & Diamond, 2000), amphibians (Olsson et al., 2025; Sundqvist & Holmgren, 2008), and fishes (Kitazawa & Kaiya, 2021; Olsson et al., 2025; Olsson & Holmgren, 2011). Figure 6 illustrates spontaneous, apparently unregulated contraction in stomach strips dissected from metamorphosing and juvenile African clawed frogs, *Xenopus laevis*, and their response to l‐NAME, which also stimulates stomach contraction in mammals (Groneberg et al., 2016) and likely other vertebrates.

**FIGURE 6:**
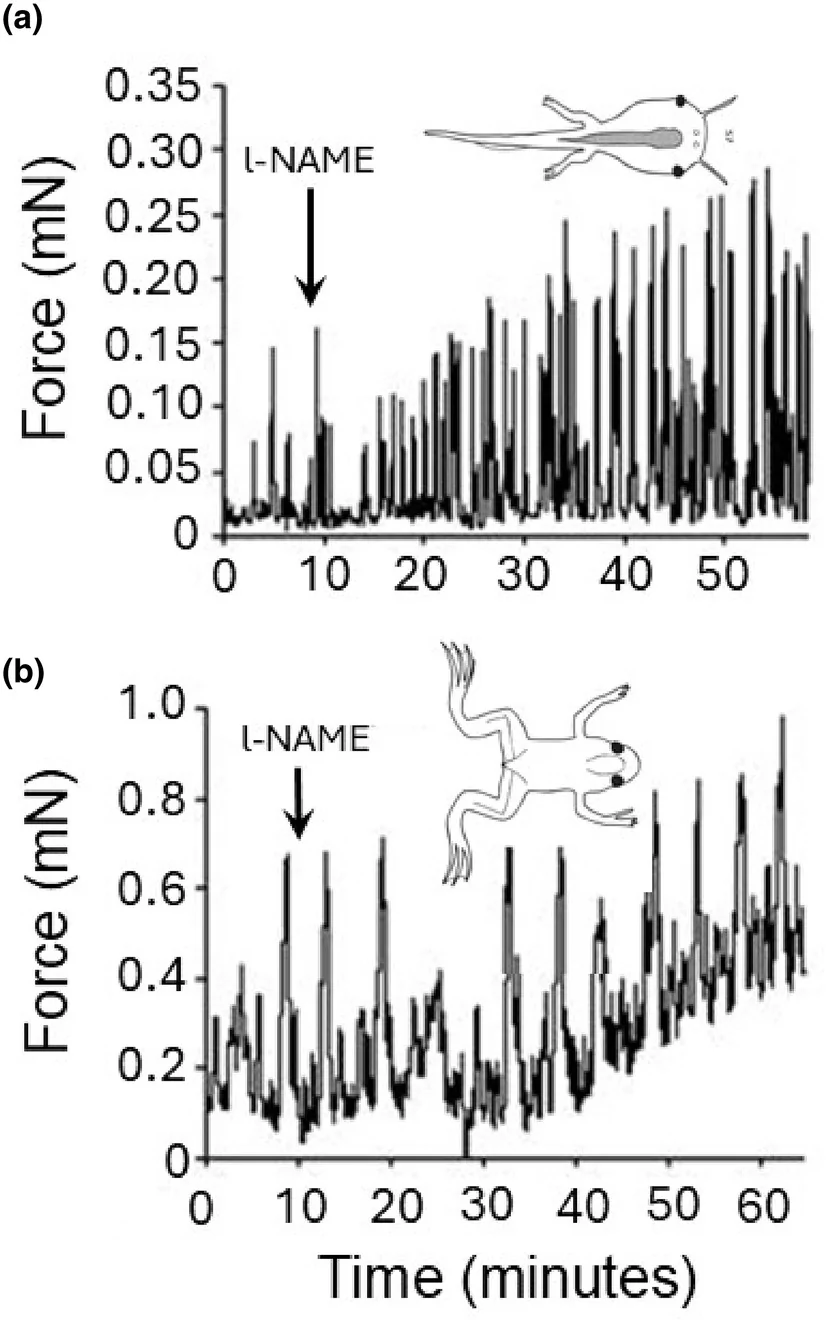
Spontaneous activity in stomach strips dissected from metamorphosing (a) and juvenile (b) African clawed frogs, Xenopus laevis. Although not directly reflecting in vivo patterns of level or activity of gastric emptying, excised stomach strips show great variation in the frequency and magnitude of spontaneous contractions following stimulation in vitro by Nω‐nitro‐L‐arginine methyl ester (l‐NAME) at arrows. Modified from Sundqvist and Holmgren (2008).

#### 3.2.5 Case study #9: Immune system T cell production

Immunological homeostasis involves immunometabolism, immune checkpoints, cytokine signaling, regulatory T and B cells and, more recently discovered, epigenetic modulation (Arneth, 2025; Gómez‐Manríquez et al., 2025; Han et al., 2025). However, even in this homeostatic framework, immune system activity can be irregular and even stochastic (Abadie et al., 2019; Fatehi et al., 2018). Immune dynamics are shaped by intrinsic noise, discrete cellular events, and fluctuating microenvironments, in combination leading to non‐deterministic, burst‐like, and variable responses rather than perfectly periodic patterns reflecting tight homeostatic regulation (Milutinovic & De Boer, 2007; Sigawi et al., 2024). Rather than reflecting complete lack of regulation, however, this immune system stochasticity actually serves an important purpose—providing the oxymoronic “stochastic regulation” of immune defenses. Rare precursor cells lead to many specialized cell types to provide “on‐demand” protection against infectious threats, for example Abadie et al. (2019). Random, infrequent intracellular regulation, as opposed to predictable “always on” regulatory mechanisms, determines immune cell differentiation and population density and composition. This allows genetically identical cells to nonetheless assume different fates under identical conditions.

Essentially, irregularity and even stochasticity in immune system activity reflects a “bet‐hedging strategy” providing adaptive flexibility and robustness to immune system defenses (Tate & Van Cleve, 2022). The immune response to an infectious pathogen serves as an example. So‐called “immune polarization”, where specialized helper T cells focus on a single pathogen species, would theoretically provide rapid, highly effective clearance of the parasite, but may inadvertently leave the host susceptible to other pathogens. This approach results in high variability in overall control against pathogen infection (Figure 7). In contrast, “immunological bet‐hedging” would generate a diverse population of T cell subtypes created by stochastic production of variable T cell subtypes. The result would be a potentially more consistent defense across an array of parasites even while sacrificing maximum efficiency against any one pathogen. It is important to emphasize that bet‐hedging results from stochastic generation of varied T cell populations. While this is ultimately a regulated process, there are by intent highly stochastic elements to T cell production.

**FIGURE 7:**
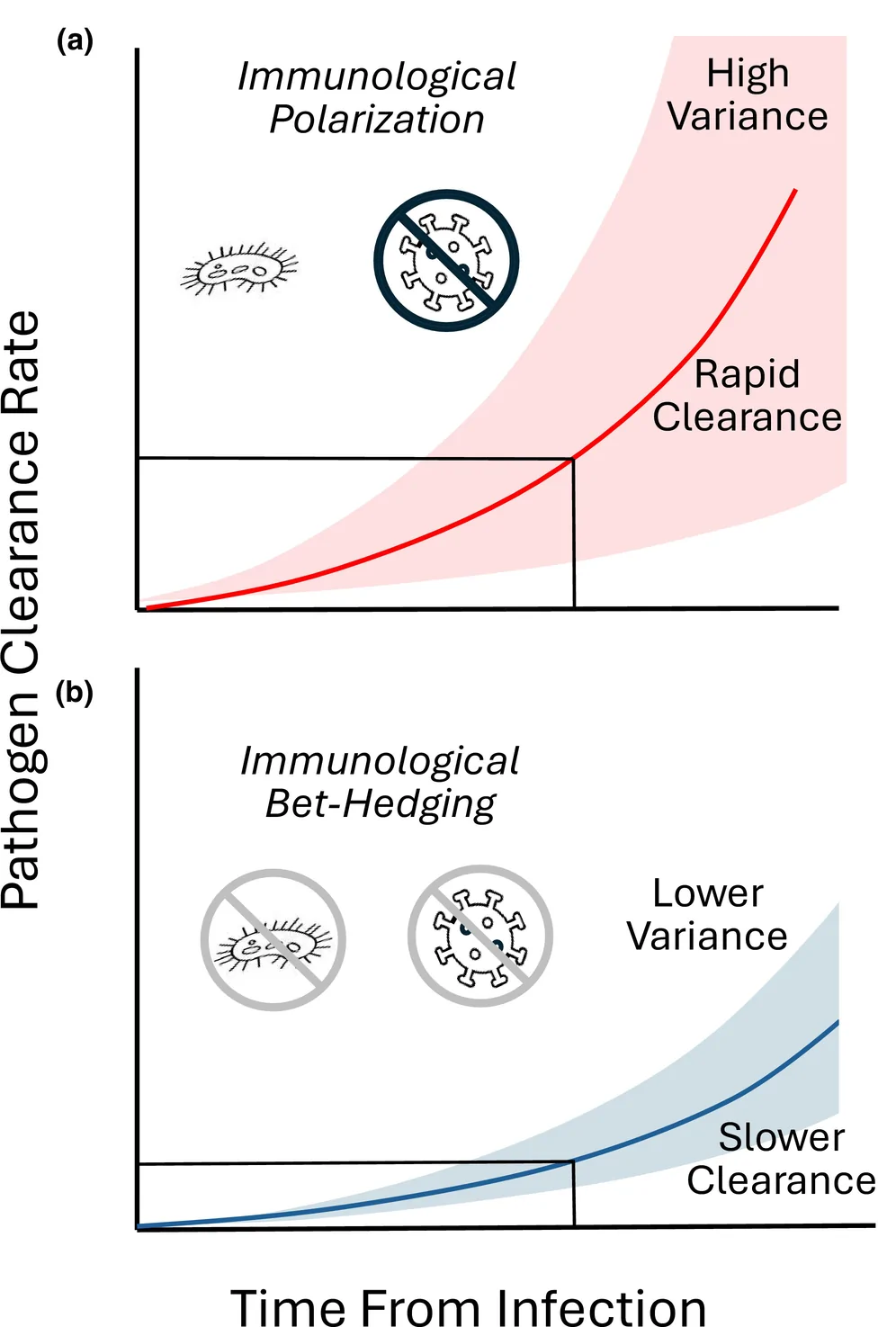
Hypothetical pathogen clearance rates under unpredictable infection conditions in an immune system with “bet‐hedging” versus “polarization” immune responses. (a) The immunological “polarization” model of T cell immunity provides very targeted and intense responses to a particular pathogen but may leave the organism vulnerable to other pathogens against which it has little or no immunity. In contrast, the bet‐hedging approach (b), resulting from the stochastic creation of a wide variety of T cells, provides moderate immunity against a specific pathogen, but concurrently some immunity against a wide range of different pathogens. Consequently, there is lower variability of the likely immune response in bet‐hedging immunity, because statistically there is some small population of a T cells that will provide some protection against infection protection. After Tate and Van Cleve (2022).

### 3.3 Homeostatic regulation, sort of……

#### 3.3.1 Case study #10: Enantiostasis as an outlier in the constellation of homeostatic concepts?

Finally, “enantiostasis” emphasizes constancy of function rather than constancy of the *milieu intérieur* (Mangum & Towle, 1977; Ruoff, 2026) (Table 1), which in many ways is an alternative to canonical homeostasis. A striking example of such constancy of function in the face of both external and internal saline stressors occurs in estuarine invertebrates like the blue crab (*Callinectes sapidus*). This species lives in estuaries where environmental salinity fluctuates greatly. As the environmental salinity decreases from 35 ppt down to ~27 ppt, hemolymph osmolality closely tracks ambient osmolality (Figure 8) (Mangum & Towle, 1977). However, as the osmolality of the surrounding water falls below 27 ppt, the hemolymph and tissues of the blue crab become ‘hyperosmotic’ as they regulate hemolymph osmolality at levels above ambient all the way down to essentially freshwater. Yet, despite this ability to maintain a hyperosmotic state, hemolymph osmolality continues to decline as ambient salinity falls, albeit at a much slower rate than the decline from ambient salinities of 35 to 27 ppt. That is, they only partially defend the *milieu intérieur*. Adult blue crabs, however, can remain fully functional for days or even weeks in freshwater (Mantel & Farmer, 1983; deFur et al., 1988). This constancy of overall function rather than constancy of *milieu intérieur* emblematic of enantiostasis is, in fact, typified by osmoregulatory patterns of several other estuarine species, including the brown shrimp *Crangon crangon*, the Dungeness crab *Cancer magister*, and the striped shore crab *Pachygrapsus crassipes*, among others (Mantel & Farmer, 1983; McNamara & Faria, 2012).

**FIGURE 8:**
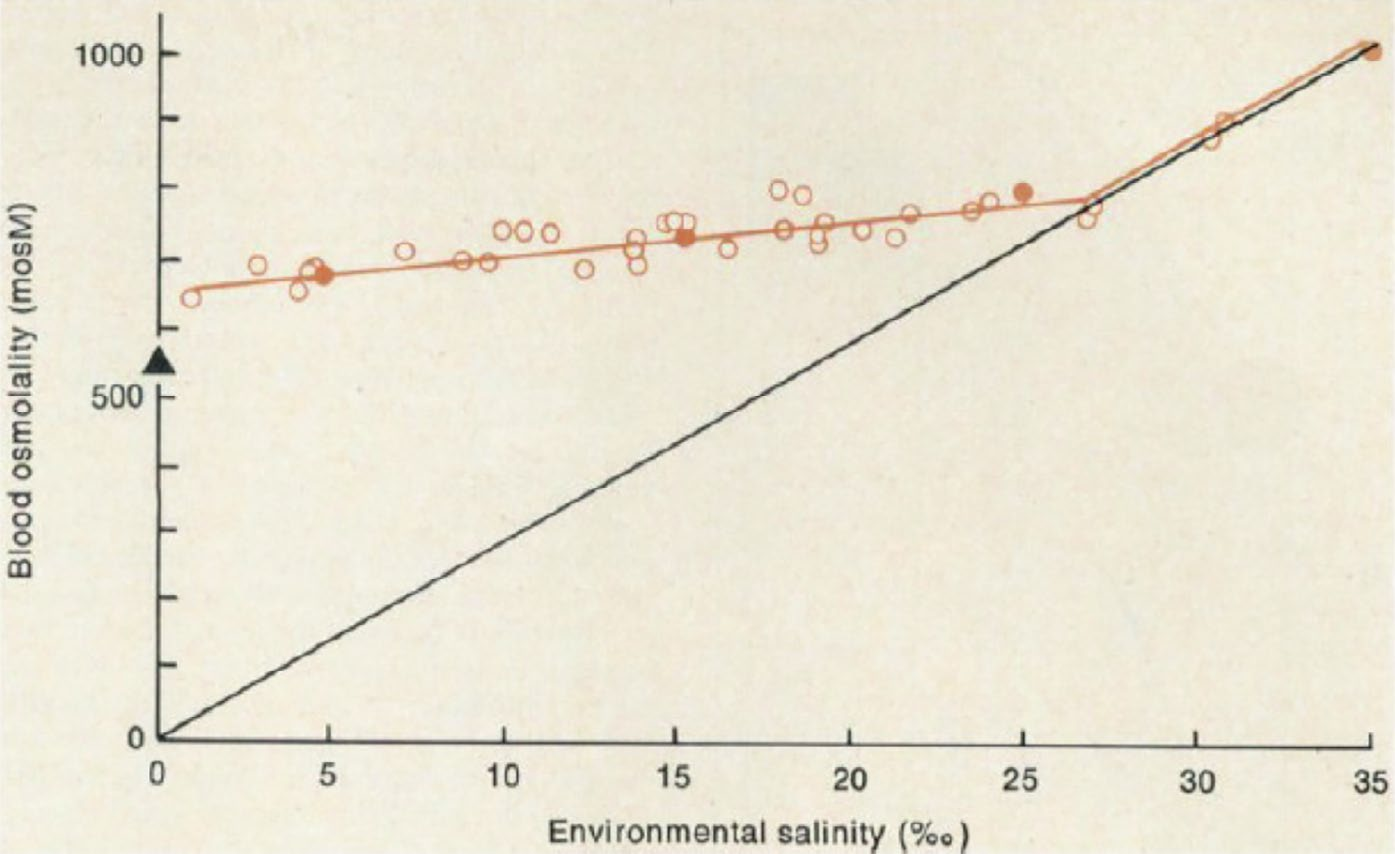
Original Figure from Mangum and Towle (1977) showing a pattern of osmoregulation in the blue crab (Calinectes sapidus) that they termed “enantiostasis”. Open and closed circles represent wild caught and laboratory acclimated crabs, respectively. Essentially, blue crabs can survive, if not thrive, across a very broad range of environmental salinities that they encounter in their estuarine habitat. As environmental salinity drops from 35 ppt to 27 ppt (x‐axis), they allow hemolymph osmolality to track environmental levels (Y‐axis), conserving energy that would have otherwise been spent on osmoregulation at the gills. However, as salinity falls still lower (perhaps from a heavy freshwater incursion into the estuary) they continue to partially—but not fully—defend hemolymph osmolality. In freshwater, where they can live but not reproduce, their hemolymph osmolality (black triangle) is only 55% of what it is typically in full strength seawater. Thus, blue crab allows hemolymph and tissue osmolalities to vary widely even while maintaining all but reproductive function. See text for additional discussion.

Another example of enantiostasis involves extracellular fluid homeostasis in insects. Most insects osmoregulate to maintain extracellular fluid volume, solute content, and osmotic pressure within fairly wide tolerances (Beyenbach, 2016; Halberg & Denholm, 2024; Koyama et al., 2023). Yet, many insects can also tolerate large changes in these parameters while generally maintaining organismal function (Beyenbach, 2016). They achieve this by a combination of “correcting” the internal changes that are occurring in extracellular fluid volume, solute content, and osmotic pressure and/or “compensating” for changes in one osmoregulatory variable by changes in one of the others.

Ultimately, it may be a rather than physiological argument as to whether enantiostasis in insects, crustaceans, or other animals is regarded as a notable outlier in the constellation of homeostatic concepts or is simply a variant on adaptive homeostasis.

## 4 HOMESTASIS IS NOT ALL‐PERVASIVE

### 4.1 Common elements in these case studies

As apparent in the many case studies presented above, canonical homeostasis co‐exists with non‐steady state and even stochastic regulation and correlated variation in physiological variables. Typically, such non‐steady state regulation does not represent a failure to regulate—that is, dysfunction or pathology. Rather, these case studies indicate that high degrees of irregular variation across tissues and time represent “business as usual” for many animals. Notably, even in humans, the mammal often showcased as exhibiting a high (highest?) degree of homeostasis, certain physiological processes can at times fall well outside of the stereotypical paradigm of homeostatic regulation (e.g., Case Studies #5–9).

Another common feature abundantly apparent from the Case Studies is that canonical homeostasis co‐exists with other forms and patterns of highly variable regulation. Thus, oxygen transport tightly regulated by the cardiorespiratory can co‐exist with pulsatile, stochastic endocrine secretion (outlined in Case Study #5). Body temperature can be tightly regulated even as production of immune system elements can be irregular or stochastic (outlined in Case Study #9). Summarizing, in any given animal at any given time, homeostatic regulation is not necessarily all‐pervasive—that is, not all body systems nor all physiological processes are regulated following the canonical homeostatic paradigm developed by offered by Claude Bernard (Cooper, 2008) and expanded and formalized by Walter Canon (Cannon, 1932). Indeed, what these case studies indicate is that it is quite common for key physiological systems that variables associated with one organ system or physiological process may be quite closely regulated within narrow limits, while simultaneously another set is more loosely regulated or even temporarily unregulated (Figure 9). This more complex view of homeostasis emerges when one isolates and focuses on particular set of regulated variables involving a system of interest, as evident from numerous case studies.

**FIGURE 9:**
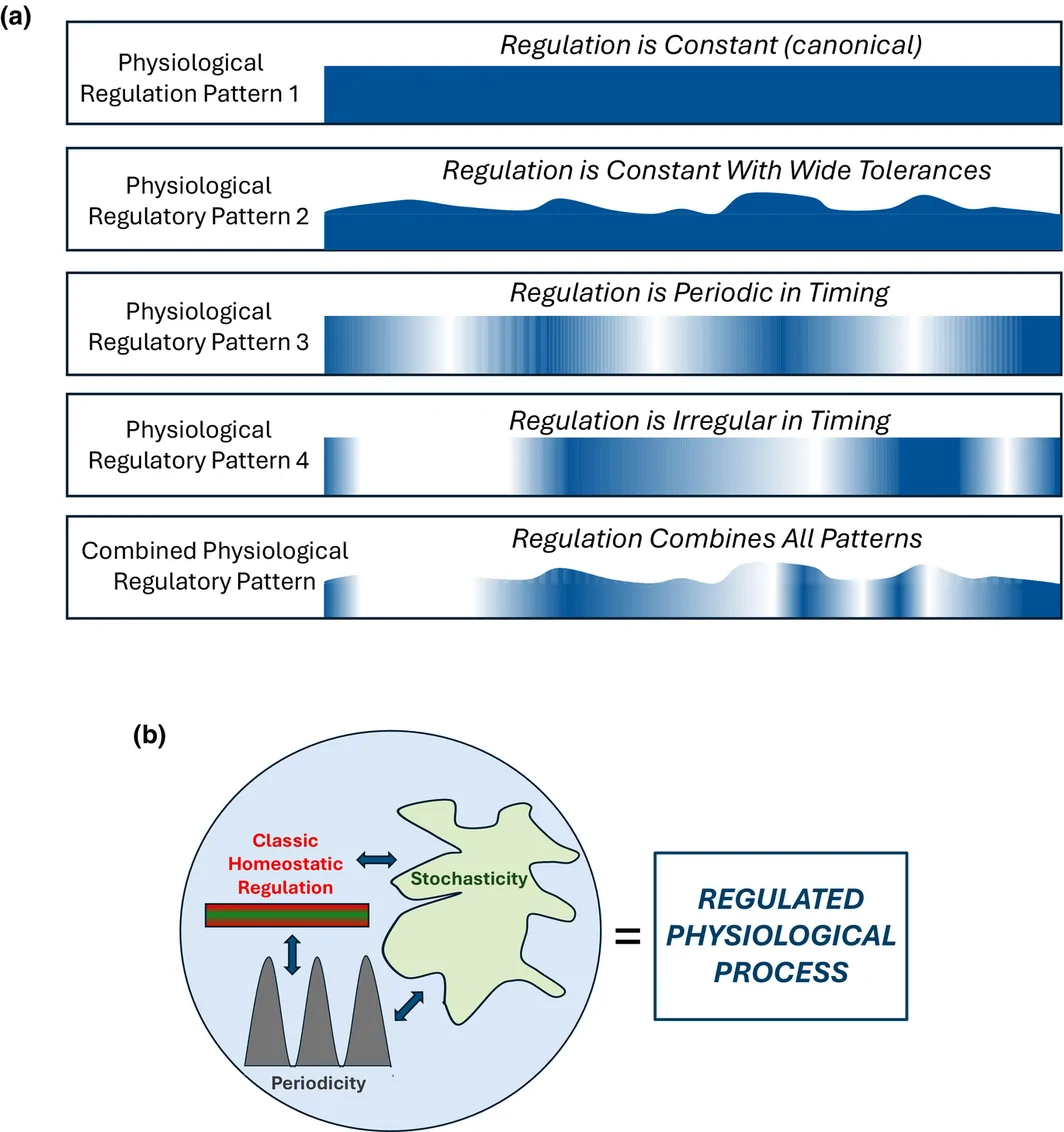
Regulation of many physiological variables can have different components acting concurrently or in series. (a) Hypothetical regulation of a physiological variable that can vary between constant, periodic, irregular of combined components. (b) A schematic summary of major components that in concert result in a regulated physiological process.

### 4.2 Energetic and evolutionary considerations

It is important to indicate that even stochastic swings in physiological variables can occur in regulatory systems, reflecting an alternative view of what comprises homeostatic regulation. Periodicity or even stochasticity has likely evolved as part of overall regulatory processes (Figure 8). Returning to hibernation as an example, investigators have concluded that during hibernation metabolic rate is actively rather than passively suppressed (Boyer & Barnes, 1999), fluid homeostasis is regulated (Junkins et al., 2024) and blood clotting is actively maintained at temperature‐appropriate levels (De Vrij et al., 2023). Homeostatic regulation also enables relatively stable, low body temperature at levels slightly above freezing (Drew et al., 2007). Yet, some hibernating animals exhibit stochastic fluctuations in temperature (“flickering”) during hibernation that are clearly outside the framework of classic homeostatic regulation—even at the low core temperatures of hibernation (Oro & Freixas, 2021). Thus, many regulatory systems regarded as typifying some form of homeostasis (Table 1) may contain hidden within irregular, imprecise, or stochastic elements.

## 5 PRACTICAL CONSIDERATIONS—FUTURE EXPERIMENTS

Falsifying the hypothesis that physiological variables are still being regulated when they are ‘permitted’ to venture out of the “normal” range and even show unpredictable, stochastic temporary patterns—let alone that they evolved because they are more energy‐efficient—is not trivial. Specific experimental criteria to separate adaptive intermittency from “simple” failure of control have yet to be identified. However, experimentation might potentially involve active intervention in normal homeostatic regulatory systems through the use of pharmaceutical agonists/antagonists (assuming the target tissue is known), surgical intervention involving cutting/stimulating nerves (again, assuming the target tissue is known), or applying environmental stressors. Following experimentally altered physiological regulation, investigators could assess, for example, how regulatory feedback gain varies with time/condition or whether there are cyclic or stochastic “programmed” “off” and “on” phases (and whether the transitions between these states are graded or abrupt). Differences in metabolic state or rate could also be measured under different conditions of regulatory intervention.

We encourage such experiments as homeostatic regulation is re‐examined and re‐interpreted.

## 6 CONCLUSIONS AND FUTURE DIRECTIONS

What emerges from taking a new angle on canonical homeostasis is that highly plastic, irregular, intermittent and even stochastic processes are very likely not the result of ineffective homeostatic regulation—that is, dysfunction or pathology. Rather, we argue that such stochastic components of regulatory systems emerged through natural selection. Determining the factors leading to the evolution of such non‐steady state and even stochastic elements is speculative, of course. However, against this backdrop, it may be that irregularity, periodicity or stochasticity in regulatory systems has evolved because the overall energetic costs of such a system are lower (and hence more likely to have been selected for) than a system that maintains canonical homeostasis.

## AUTHOR CONTRIBUTIONS

**Warren Burggren:** Conceptualization; validation; visualization. **Tobis Wang:** Conceptualization.

## FUNDING INFORMATION

This study was supported by grant #2103499 from the National Science Foundation to WB.

## CONFLICT OF INTEREST STATEMENT

The author declares no conflict of interest.

## ETHICS STATEMENT

Ethical approval was not required for this review, which is based on previously published literature and does not report any new studies involving human participants or animals.

## PERMISSION TO REPRODUCE MATERIAL FROM OTHER SOURCES

Permission has been obtained for reproduction of figures from other sources.
